# Role of Paricalcitol in Modulating the Immune Response in Patients with Renal Disease

**DOI:** 10.1155/2015/765364

**Published:** 2015-09-15

**Authors:** Silvia Lucisano, Adriana Arena, Giovanna Stassi, Daniela Iannello, Gaetano Montalto, Adolfo Romeo, Giuseppe Costantino, Rosaria Lupica, Valeria Cernaro, Domenico Santoro, Michele Buemi

**Affiliations:** ^1^Chair of Nephrology, Department of Clinical and Experimental Medicine, University of Messina, 98124 Messina, Italy; ^2^Department of Human Pathology, Unit of Clinical Microbiology, University of Messina, 98124 Messina, Italy

## Abstract

*Introduction*. The aim was to highlight the existence of a relationship between vitamin D deficiency, chronic inflammation, and proteinuria, by measuring neutrophil gelatinase associated lipocalin (NGAL) and common inflammatory markers after administration of paricalcitol, a vitamin D analog, * in vivo* and *in vitro*. *Methods*. 40 patients with end-stage chronic kidney disease (CKD) and secondary hyperparathyroidism and 40 healthy subjects were enrolled. Serum calcium, phosphorus, 25(OH)-vitamin D, parathyroid hormone (PTH), erythrocyte sedimentation rate, high-sensitivity C-reactive protein, interleukin- (IL-) 17, IL-6, IL-1*β*, interferon-gamma (IFN-*γ*), tumor necrosis factor-alpha (TNF-*α*), plasmatic and urinary NGAL, and 24 h albuminuria and proteinuria were measured before and 24 h after an intravenous bolus of paricalcitol (5 mcg). Human peripheral blood mononuclear cells were isolated and stimulated with phytohaemagglutinin. NGAL, IL-1*β*, IL-17, IL-6, TNF-*α*, and IFN-*γ* were measured in the culture medium and in the 24 h urine collection. *Results*. 25(OH)-vitamin D was lower in CKD than in controls (*p* < 0.0001), while inflammatory markers were higher in CKD group (*p* < 0.0001). *In vivo* and *in vitro* studies showed a downregulation of NGAL, IL-17, IL-6, IL-1*β*, TNF-*α*, and IFN-*γ* after paricalcitol administration (*p* < 0.0001). *Conclusions*. 25(OH)-vitamin D regulates immune and inflammatory processes. Further studies are needed to confirm these data in order to improve the treatment of CKD patients.

## 1. Introduction

Microinflammation state is a pathologic feature of chronic kidney diseases (CKD). Renal inflammation, characterized by the infiltration of inflammatory cells including T cells and macrophages and the release of proinflammatory and chemoattractant cytokines to kidney parenchyma, is a critical process leading to progression of disease in CKD patients.

Vitamin D has been shown to have potent anti-inflammatory effects and, consequently, has been considered for adjunctive therapy in the treatment of numerous chronic diseases including asthma, rheumatoid arthritis, multiple sclerosis, diabetes mellitus type 1, psoriasis, chronic inflammatory bowel diseases, and prostate cancer [1–3]. A variety of pro- and anti-inflammatory effects for vitamin D have previously been reported [4, 5].

Several lines of evidence have suggested a potential anti-inflammatory activity of 1,25 (OH)2 vitamin D3 and vitamin D analogs in CKD [6–9]. In animal models of primary glomerular diseases, administration of paricalcitol or 1,25 (OH)2 vitamin D3 reduces glomerular infiltration of inflammatory cells [6, 10]. Consistently, a reduced inflammation is associated with higher serum 25(OH)-vitamin D levels in patients with CKD [11].

Evidence is also mounting that paricalcitol, a synthetic vitamin D analog, is renoprotective in different experimental nephropathies [6, 12]. The effect of paricalcitol on renal inflammation was investigated in a mouse model of obstructive nephropathy. Paricalcitol reduced infiltration of T cells and macrophages in the obstructed kidney. Moreover, the benefits are greater with the newer vitamin D analogs, paricalcitol or doxercalciferol, when compared with calcitriol [13, 14].

The mechanism by which vitamin D and its analogs reduce inflammation remains poorly understood.

It is possible that 1,25(OH)2 vitamin D3 may exert its immunomodulatory action through regulating the activity of many types of immune cells such as macrophages, dendritic cells, and T cells [15, 16].

An important marker of inflammation in CKD patients is neutrophil gelatinase associated lipocalin (NGAL), a 25-kDa glycoprotein first found involved in a variety of cellular processes, including the innate immune response [17–20]. NGAL expression is also found in epithelial cells and it is strongly and rapidly induced in the nephrons in the presence of inflammation and in response to renal epithelial injury [21–25].

In this study we evaluated the anti-inflammatory action of paricalcitol in CKD patients with secondary hyperparathyroidism. For this purpose, we measured the common inflammatory markers, some hallmarks of Th-1 (IFN-*γ*, IL-1*β*, and TNF-*α*), Th-2 (IL-6), and Th-17 (IL-17) response, as well as NGAL production before and after a single intravenous administration of paricalcitol* in vivo* and* in vitro*.

## 2. Materials and Methods

*Patients and Controls*. The study series consisted of 40 patients with CKD stages 4 and 5 and secondary hyperparathyroidism [20 men (50%) mean age 62.9 ± 14 y]. Patients with inflammatory disease, cancer, poorly controlled diabetes mellitus (hemoglobin A1c of >11%), hyperphosphatemia (>6.5 mg/dL), or hypercalcemia (>10.5 mg/dL) were excluded from the study.

Calcitriol therapy in CKD patients was suspended five weeks before the study.

The causes of renal failure were diabetes (*n* = 20), nephroangiosclerosis (*n* = 8), chronic glomerulonephritis (*n* = 4), polycystic kidney disease (*n* = 2), and other causes (*n* = 6).

Control group consisted of 20 healthy subjects (HS) [10 men and 10 women, mean age 54.8 ± 4.8 y].

The study was approved by the local ethics committee and fully informed consent was obtained from all participants.

### 2.1. *In Vivo*

*Management of Blood Samples.* CKD patients were hospitalized; blood samples were collected in the morning at 08.00 h before and after 24 hours of a single intravenous administration of 5 mcg of paricalcitol (Zemplar, Abbott s.r.l., Italy).

Blood samples were collected into chilled vacutainer tubes containing potassium ethylenediamine tetraacetate. Tubes were instantly cooled on ice and centrifuged at 3000 rpm for 10 min at 4°C within 30 min. Plasma was stored at −80°C until analyzed. As a control, we analyzed plasma samples of healthy donors. Furthermore, we collected blood samples from CKD patients and HS in order to obtain sera to measure biochemical parameters.

At 08:30 am we administered a single intravenous dose of paricalcitol (5 mcg) to CKD patients.

We decided to use intravenous and not oral administration of paricalcitol to prevent deficient absorption of the drug due to gastrointestinal disorders.

We measured biochemical parameters, including serum levels of urea, creatinine, calcium, phosphorus, PTH, albuminuria 24 hours, proteinuria 24 hours, erythrocyte sedimentation rate (ESR), 25(OH)-vitamin D levels, and high sensitivity C-reactive protein (hsCRP), according to standard methods in the routine clinical laboratory.

Plasmatic and urinary NGAL levels and plasmatic IL-17, IL-6, IL-1*β*, TNF-*α*, and IFN-*γ* levels were measured using a commercially available ELISA kit (R&D system, Milan, Italy).

The same determinations were made before and after 24 h of intravenous paricalcitol administration (5 mcg).

### 2.2. *In Vitro*

*Isolation of Human Peripheral Blood Mononuclear Cells (PBMC).* PBMC were isolated from heparinized, venous blood of patients affected by CKD 24 hours before and after paricalcitol administration.

PBMC, after centrifugation over Ficoll-Hypaque gradient, were then washed three times in RPMI 1640 medium (Sigma) and cultured in 24-well plates at a concentration of 2 × 10^6^ cells/mL per well in RPMI 1640 medium. PBMC were cultured at 37°C in 5% CO_2_ atmosphere, in RPMI 1640 supplemented with 50 *μ*g/mL gentamicin and 5% fetal calf serum (FCS, Sigma). All reagents were supplied by Sigma Aldrich (Milan, Italy).

*Treatments of PBMC.* Phytohaemagglutinin A (PHA), used as a stimulator of PBMC at a concentration of 10 *μ*g/mL, was supplied by Sigma Aldrich (Milan, Italy).

48 hours after treatment, the supernatants were harvested, and suitable aliquots were stored at –80°C until cytokine analysis.

*Limulus Test.* Culture media and reagents tested for the presence of endotoxin using the E-Toxate kit (Sigma, Milan) were found to contain <10 pg of endotoxin per mL.

*Cytokine Evaluations.* Supernatants from PBMC in different experimental conditions were harvested, centrifuged, and kept at −80°C until titration for the presence of IL-17, IL-6, IFN-*γ*, IL-1*β*, TNF-*α*, and NGAL by an immunoenzymatic method (ELISA); the kits used were supplied by R&D system (Milan, Italy) and NGAL (BioPorto Diagnostics, Verona, Italy), respectively. The minimum detectable dose of IL-17 was less than 15 pg/mL, of IL-6 less than 1.4 pg/mL, of IFN-*γ* less than 1.5 pg/mL, of IL-1*β* less than 1 pg/mL, of TNF-*α* less than 1.6 pg/mL, and of NGAL less than 0.1 ng/mL.

### 2.3. Statistical Analysis

Statistical analyses were performed with NCSS for Windows (version 4.0), the MedCalc (version 8.0) software, and the GraphPad Prism (version 5.0) package. Data were presented as mean ± SD for normally distributed values (at Kolmogorov-Smirnov test) and median [IQ range] for nonnormally distributed values. Differences between groups were established by unpaired *t* test for normally distributed values and by Kruskal-Wallis analysis followed by Dunn's test for nonparametric values. Spearman's correlation coefficient was calculated to examine the relation between variables. Before testing correlations, all nonnormally distributed values were log-transformed to better approximate normal distributions. Stepwise multiple regression analyses were performed in order to assess independent relationships. All results were considered significant if *p* was < 0.05.

## 3. Results

### 3.1. *In Vivo*

The main characteristics of the study cohort are summarized in Table 1. The mean age of CKD patients was 62.9 ± 14 years.

The calcium/phosphorus metabolism was significantly altered in CKD subjects compared with HS, total serum calcium levels being lower and phosphorus and the serum CaxP product higher. At baseline, 25(OH)-vitamin D levels were low in CKD patients: all of our study participants had baseline 25(OH)-vitamin D levels <20 ng/mL, and 70% had a level <15 ng/mL. PTH levels were significantly higher in CKD patients compared with healthy controls (*p* < 0.0001).

Inflammatory markers, such as ESR and hsCRP, were higher than values in healthy controls (*p* < 0.0001). Sixty percent of our patients had an elevated baseline hsCRP (>2.5 mg/L), while seventy percent had ESR values >30 mm/h. Also baseline plasmatic and urinary NGAL values and plasmatic inflammatory cytokines (IL-17, IL-6, IL-1*β*, TNF-*α*, and IFN-*γ*) in CKD patients were significantly higher than in healthy subjects.

After intravenous paricalcitol administration, there was a significant increase in 25(OH)-vitamin D levels (*p* < 0.0001) associated with a reduction of PTH, but not in a statistically significant way (*p* = 0.2).

Calcium and phosphorus levels and CaxP product did not change in significant way.

**Figure 1:**
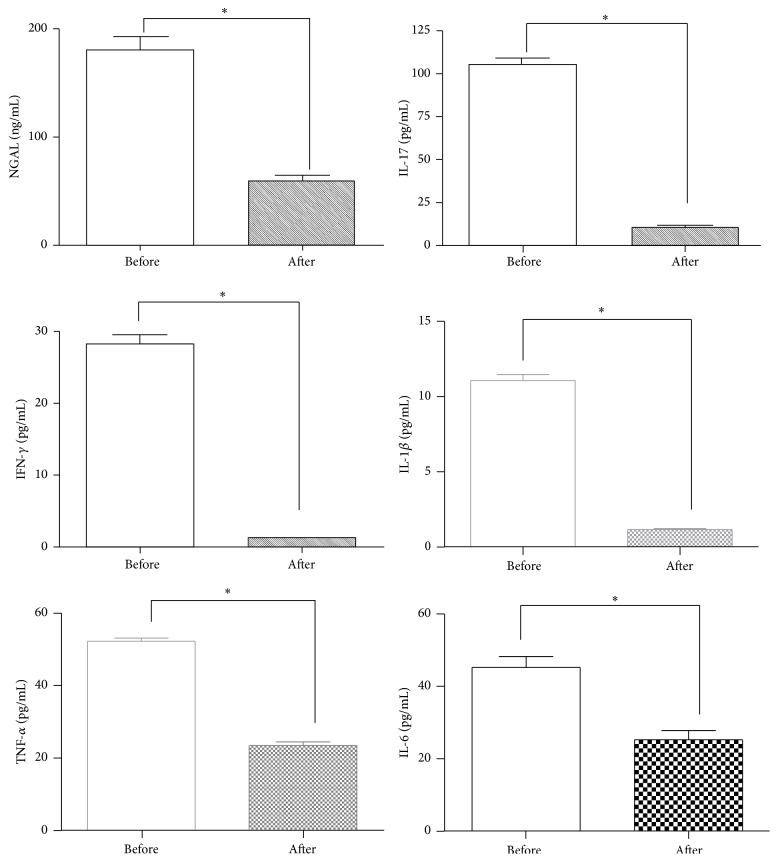
Values of serological parameters, before and after paracalcitol supplementation. A significant reduction (*p* < 0.0001) in NGAL values and in cytokine levels (IL-17, IFN-gamma, IL-1 beta, TNF-alfa, and IL-6) occurred from plasma samples obtained from all CKD patients ^*∗*^ *p* < 0.0001.

ESR values were reduced after paricalcitol in a statistically significant way (*p* = 0.02); hsCRP values were also reduced, but not in a significant way (*p* = 0.3). After paricalcitol supplementation, a significant reduction (*p* < 0.0001) in NGAL values and in cytokine levels occurred in plasma samples obtained from CKD patients, Figure 1.

We observed a slight reduction in the levels of albuminuria 24 h and proteinuria 24 h in CKD patients after paricalcitol, but not in a significant way (*p* = 0.1).

### 3.2. *In Vitro*

PBMC stimulation induced a significant NGAL upregulation and cytokine production, both in uremic patients and HS compared with unstimulated PBMC.

In CKD patients after paricalcitol supplementation, there was a significant reduction of NGAL and inflammatory cytokine production by stimulated PBMC (*p* < 0.0001), returning to values similar to those of healthy subjects.

Table 2 summarizes those results.

*Univariate Correlations for Paricalcitol.* At univariate analysis,* in vivo* NGAL was found to be directly correlated with ESR (*r* = 0.35; *p* = 0.02), IL-17 (*r* = 0.64; *p* < 0.0001), IL-6 (*r* = 0.90; *p* < 0.0001), IFN-*γ* (*r* = 0.90; *p* < 0.0001), IL-1*β* (*r* = 0.51; *p* = 0.0007), TNF-*α* (*r* = 0.59; *p* < 0.0001), and hsPCR (*r* = 0.81; *p* < 0.0001).

*In vitro* NGAL was found to be directly correlated with ESR (*r* = 0.35; *p* = 0.02), hsPCR (*r* = 0.36; *p* = 0.002), IL-17 (*r* = 0.97, *p* < 0.0001), IL-6 (*r* = 0.94, *p* < 0.0001), IL-1*β* (*r* = 0.97; *p* < 0.0001), and TNF-*α* (*r* = 0.96; *p* < 0.0001), while an inverse correlation was found with 25(OH)-vitamin D levels (*r* = −0.48; *p* = 0.001).

In contrast, no significant correlation was found for other parameters such as albuminuria, proteinuria 24 h, PTH, calcium, or phosphorus (*r* range from 0.42 to 0.12; *p* range from 0.82 to 0.27).

*Multiple Regression Analysis.* All variables found to be significantly correlated with NGAL at univariate analysis were introduced in a multivariate model using NGAL as a dependent variable.* In vivo*, after adjustment for other factors, significance was maintained for the correlation between NGAL and ESR (*β* = 0.32; *p* = 0.001), hsCRP (*β* = 0.81; *p* < 0.0001), IL-17 (*β* = 0.32; *p* = 0.006), IL-6 (*β* = 0.78; *p* < 0.0001), and IFN-*γ* (*β* = 0.75; *p* < 0.0001) and TNF-*α* (*β* = 0.76; *p* < 0.0001).

*In vitro* significance was maintained for the correlation between NGAL and 25(OH)-vitamin D (*β* = −0.28, *p* = 0.001) and IL-1*β* (*β* = 0.08, *p* = 0.001), IL-6 (*β* = 0.59, *p* = 0.0001), and TNF-*α* (*β* = 0.43, *p* = 0.002). In contrast, the correlation with ERS, hsCRP, and IL-17, found at univariate analysis, was lost.

Table 3 summarizes the data obtained.

## 4. Discussion

Aside from its classical role as a modulator of calcium metabolism and bone health, vitamin D has been shown to have potent anti-inflammatory effects and, consequently, has been considered for adjunctive therapy in the treatment of numerous chronic diseases including asthma, arthritis, and prostate cancer [1, 3]. A variety of pro- and anti-inflammatory effects for vitamin D have previously been reported [5].

We showed the acute effects of a single intravenous administration of paricalcitol on inflammation* in vivo* and* in vitro*. For this purpose, we used NGAL, IL-17, IL-6, IL-1*β*, TNF-*α*, and IFN-*γ* as inflammatory markers. In recent years, 25(OH)-vitamin D deficiency in humans has received significant attention in CKD patients [26]. Moreover, VDR gene polymorphisms have been shown to be associated with left ventricle hypertrophy in patients with renal diseases [27].

Our results demonstrate that paricalcitol possesses an anti-inflammatory activity, resulting in a significant reduction of inflammatory markers and proinflammatory cytokines in patients with advanced CKD.

After acute paricalcitol supplementation, there is a significant reduction in plasmatic and urinary NGAL values and in IL-17, IL-6, IL-1*β*, TNF-*α*, and IFN-*γ* levels in CKD patients; values return to a normal range.

It is known that 1,25(OH)2 vitamin D3 modulates the immune system by determining direct regulatory effects on the functions of B and T lymphocytes and influencing the phenotype and function of the antigen presenting cells (APC) and dendritic cells (DC), promoting properties that favor the induction of tolerogenic T regulators rather than T effectory [28]. This adjustment is mediated by the action of 1,25(OH)2D3 on nuclear transcription factors, such as NF-AT and NF-*κ*B or direct interaction with VDRE in the promoter regions of the genes of cytokines. The vitamin is produced by the macrophages and by DC, T, and B cells, and is, therefore, capable of contributing physiologically, through VDR expressed in their nucleus, to the regulation of innate, rather than adaptive, autocrine and paracrine immunity [28, 29]. Vitamin D also enhances the response of the innate immune system through activation of toll-like receptor (TLR) [30, 31].

Our results confirm vitamin D immunomodulatory action, which is conducted by effectively inhibiting proinflammatory cytokines (IL-17, IL-6, IL-1*β*, TNF-*α*, and IFN-*γ*) produced* in vitro* by PBMC.

*In vivo* basal production of IL-17, IL-6, IL-1*β*, IFN-*γ*, TNF-*α*, and NGAL is increased in CKD patients compared to healthy controls.

*In vitro* the PBMC produced a significant upregulation of NGAL, IL-17, IL-6, IL-1*β*, TNF-*α*, and IFN-*γ* in both uremic patients and HS. Paricalcitol supplementation determined a significant reduction of NGAL and inflammatory cytokine production by PBMC in CKD patients.

Thus, the anti-inflammatory effects of paricalcitol appear to be mediated by non-PTH mechanisms.

As already mentioned, our study confirmed calcium-phosphorus metabolism alterations in CKD patients, but PTH did not vary in a significant way and no correlation was found between inflammatory indices and values of PTH, calcium, or phosphorus* in vivo* or* in vitro*.

After intravenous paricalcitol administration associated with a significant increase in 25(OH)-vitamin D levels, there was a reduction of PTH levels, but not in a statistically significant way. Probably a single acute administration of paricalcitol is not sufficient to determine a significant reduction of PTH levels but, by VDR binding, increases the amount of free and measurable 25(OH)-vitamin D in circulation.

A recent study has found that human monocytes are capable of responding to treatment with two different forms of vitamin D: 1,25(OH)2 vitamin D3 and 25(OH) vitamin D3. 25(OH) vitamin D3 is converted into a functionally active form, 1,25(OH)2 vitamin D3, by the enzyme 25-hydroxyvitamin D3-1a-hydroxylase (CYP27b1), a process that primarily occurs in the kidneys [32, 33]. However, it has been shown that monocytes, macrophages, and dendritic cells also express CYP27b1 [30, 34]. Therefore, 1,25(OH)2 vitamin D3 can be produced locally and exerts immunomodulatory effects [35].

Moreover, Zhang et al. demonstrated that 15 ng/mL 25(OH) vitamin D3 [a concentration amount considered as vitamin D deficiency in this study] did not suppress LPS-induced cytokine (IL-6 and TNF-*α*) production in human monocytes. Conversely, they found that 25(OH) vitamin D3 at 30 ng/mL (a level considered to be sufficient in humans) significantly inhibited cytokine production induced by LPS,* in vitro* [36].

These data support the hypothesis that, to achieve optimal anti-inflammatory effects by vitamin D, it is important to maintain serum 25(OH)-vitamin D levels at 30 ng/mL in the physiologic range [37].

At baseline, 25(OH)-vitamin D levels were low in CKD patients: all of our study participants had baseline 25(OH)-vitamin D levels <20 ng/mL, and 70% had a level <15 ng/mL, which is not surprising as 1,25(OH)2 vitamin D3 deficiency occurs early in the course of kidney disease, given that the renal tubule is the site of active vitamin D3 synthesis [38].

After intravenous paricalcitol administration, there was a significant increase in 25(OH)-vitamin D levels. This increased production was accompanied by a highly significant reduction of the production of proinflammatory cytokines.

The low levels of 25(OH)-vitamin D may, therefore, favour the onset and the maintenance of chronic inflammation typical of our patients.

Current studies suggest that patients with chronic inflammatory diseases, who are vitamin D deficient (20 ng/mL), may benefit from oral supplementation to achieve serum 25(OH) vitamin D3 levels >30 ng/mL [36]. Additionally, active vitamin D may delay the progression of CKD [39].

In conclusion, our findings demonstrate reduction in inflammation after acute treatment with paricalcitol in patients with CKD and also confirm the involvement of this hormone in mineral metabolism. Furthermore, these benefits were not attributable to improvement in PTH.

In view of its important anti-inflammatory and immunomodulatory role, vitamin D can be used for therapeutic purposes in patients with CKD in addition to its specific role for secondary hyperparathyroidism. It is important to maintain serum levels of 25(OH)-vitamin D ≥ the value of 30 ng/mL in order to prevent the onset of subclinical inflammation typical of the terminal stages of CKD. It can also reduce the risk of cardiovascular diseases and proteinuria, especially as a preventive drug in early-stage patients.

The results of the present study show not only the appearance of new anti-inflammatory options offered by vitamin D, but also a new avenue of investigation on the effects of this vitamin on the complex cytokine network, with implications that go beyond the contrast of anti-inflammatory response. Indeed, vitamin D exerts direct effects on T cells and modifies their response to activation, thereby playing a key role in adaptive immune responses. The anti-inflammatory effects of vitamin D would consequently allow clinicians to intervene in diseases resulting from inappropriate type 1 immune responses.

## Figures and Tables

**Table 1:** Baseline demographic, clinical, and laboratory data of the study population.

Parameter	CKD patients (*n*: 40)	HS (*n*: 20)	*p* value
Gender (M/F)	20/20	10/10	—
Age (years)	62.9 ± 14	54.8 ± 4.8	**0.003**
Albumin (g/dL)	3.8 ± 6	4.09 ± 0.40	**0.02**
Calcium (mg/dL)	8.7 ± 0.7	9.15 ± 0.5	**0.04**
Phosphate (mg/dL)	5.63 ± 1.22	3.4 ± 0.35	**<0.05**
Ca × P product (mg^2^/dL^2^)	50.58 ± 11.8	33.4 ± 6.3	**<0.05**
ALPh (IU/L)	80.5 (63–110)	71.5 ± 17	0.27
PTH (pg/mL)	92.8 (33.35–85.75)	44 (11.1–15.5)	**<0.05**
hsCRP (mg/L)	3.66 (2.0–5.37)	0.49 (0.1–0.69)	**<0.05**
ESR (mm/h)	34.8 ± 8.4	4.5 ± 2.3	**<0.05**
Vitamin D (ng/mL)	11.67 ± 9.89	51.13 ± 0.4	**<0.05**
Urea (mg/dL)	234.40 (176.74–274.32)	35.6 ± 9.9	**<0.05**
Serum creatinine (mg/dL)	7.2 ± 1.9	0.75 ± 1.2	**<0.0001**
NGAL (ng/mL)	180.43 ± 86.50	29.78 ± 25.8	**<0.0001**
IL-17 (pg/mL)	105.9 ± 17.2	10.54 ± 13	**<0.0001**
IL-6 (pg/mL)	45.7 ± 10.6	15.9 ± 6.9	**<0.05**
IL-1*β* (pg/mL)	11.71 ± 3.96	1.2 ± 0.8	**<0.0001**
TNF-*α* (pg/mL)	52.56 ± 8.92	13.1 ± 2.6	**<0.05**
IFN-*γ* (pg/mL)	28.3 ± 8.5	0.98 ± 0.6	**<0.0001**
Erythrocytes (*n* × 10^6^)	3.36 ± 0.46	4.75 ± 0.26	**<0.05**
Hemoglobin (g/dL)	10.20 ± 1.33	13.46 ± 0.6	**<0.05**
Ferritin (ng/mL)	287 (150–437)	176 (163–189)	**0.01**

CKD: chronic kidney disease; HS: healthy subjects; hsCRP: high-sensitivity C-reactive protein; ALPh: alcaline phosphatase; ESR: erythrocyte sedimentation rate; NGAL: neutrophil gelatinase associated lipocalin; IL-17: interleukin-17; IL-6: interleukin-6; IL-1*β*: interleukin-1 beta; IFN-*γ*: interferon-gamma; TNF-*α*: tumor necrosis factor-alpha.

**Table:** (a) CKD patients

	Before paricalcitol		After paricalcitol	
	PBMC + PHA	PBMC	PBMC + PHA	PBMC
	(10 *μ*g/mL)		(10 *μ*g/mL)	
IL-17 (pg/mL)	39 ± 6.2	<15	19 ± 3.8	<15
IL-6 (pg/mL)	66 ± 9.8	<1.4	31 ± 5.9	<1.4
IFN-*γ* (pg/mL)	174 ± 32.9	<1.5	78 ± 11.7	<1.5
IL-1*β* (pg/mL)	97 ± 13.3	<1	42 ± 4.3	<1
TNF-*α* (pg/mL)	202 ± 31.4	<1.6	104 ± 15.8	<1.6
NGAL (ng/mL)	78 ± 9.2	<0.1	21 ± 4.1	<0.1

**Table:** (b) HS

	PBMC + PHA	PBMC
	(10 *μ*g/mL)	
IL-17 (pg/mL)	21 ± 2.9	<15
IL-6 (pg/mL)	32 ± 4.2	<1.4
IFN-*γ* (pg/mL)	59 ± 7.9	<1.5
IL-1*β* (pg/mL)	43 ± 7.1	<1
TNF-*α* (pg/mL)	97 ± 13.9	<1.6
NGAL (ng/mL)	16 ± 3.1	<0.1

CKD: chronic kidney disease; HS: healthy subjects; NGAL: neutrophil gelatinase associated lipocalin; IL-17: interleukin-17; IL-6: interleukin-6; IL-1*β*: interleukin-1 beta; IFN-*γ*: interferon-gamma; TNF-*α*: tumor necrosis factor-alpha.

**Table:** (a) *In vivo*

Variable	Partial *R*	*β*	*p* value
ESR	**0.35 (*p *** = .02**)**	**0.32**	**0.001**
hs-CRP	**0.81 (*p ***<0.0001**)**	**0.81**	**<0.0001**
IL-17	**0.64 (*p ***<0.0001**)**	**0.32**	**0.006**
IL-6	**0.90 (*p ***<0.0001**)**	**0.78**	**<0.0001**
IFN-*γ*	**0.90 (*p ***<0.0001**)**	**0.75**	**<0.0001**
TNF-*α*	**0.59 (*p ***<0.0001**)**	**0.76**	**<0.0001**
IL-1*β*	**0.51 (*p *** = 0.0007**)**	0.23	0.13

**Table:** (b) *In vitro*

Variable	Partial *R*	*β*	*p* value
ESR	**0.35 (*p *** = 0.02**)**	0.75	0.4
hs-CRP	**0.36 (*p *** = 0.002**)**	0.35	0.3
IL-17	**0.97 (*p ***<0.0001**)**	0.41	0.07
IL-6	**0.94 (*p ***<0.0001**)**	**0.59**	**0.0001**
TNF-*α*	**0.96 (*p ***<0.0001**)**	**0.43**	**0.0002**
IL-1*β*	**0.97 (*p ***<0.0001**)**	**0.08**	**0.001**
Vitamin D	−**0.48 (*p *** = 0.001**)**	**−0.28**	**0.001**

Dependent variable: NGAL; *β*: standardized coefficient of correlation.

NGAL: neutrophil gelatinase associated lipocalin; IL-17: interleukin-17; IL-6: interleukin-6; IL-1*β*: interleukin-1 beta; IFN-*γ*: interferon-gamma; TNF-*α*: tumor necrosis factor-alpha; hsCRP: high-sensitivity C-reactive protein; ESR: erythrocyte sedimentation rate.
